# Barriers to Long-Term Adherence in Botulinum Toxin Therapy for Post-Stroke Spasticity: Insights and Implications from a Single-Center Study in North Italy

**DOI:** 10.3390/toxins17030102

**Published:** 2025-02-22

**Authors:** Ester Cecchella, Nicola Luigi Bragazzi, Filippo Cotellessa, William Campanella, Luca Puce, Lucio Marinelli, Antonio Currà, Cristina Schenone, Laura Mori, Carlo Trompetto

**Affiliations:** 1 IRCCS Ospedale Policlinico San Martino, 16132 Genoa, Italy; estercecchella@gmail.com (E.C.); william.campanella@hsanmartino.it (W.C.); lucio.marinelli@unige.it (L.M.); morilaurab@gmail.com (L.M.); ctrompetto@neurologia.unige.it (C.T.); 2 Department of Neuroscience, Rehabilitation, Ophthalmology, Genetics, Maternal and Child Health (DINOGMI), University of Genoa, 16132 Genoa, Italy; filippo_cotellessa@hotmail.it (F.C.); crischenone92@gmail.com (C.S.); 3 Academic Neurology Unit, A. Fiorini Hospital, 04019 Terracina, Italy; antonio.curra@uniroma1.it; 4 Department of Medico-Surgical Sciences and Biotechnologies, “Sapienza” University of Rome, 00189 Rome, Italy

**Keywords:** stroke, spasticity, botulinum toxin, treatment discontinuation, survival analysis, Kaplan–Meier analysis, Cox regression

## Abstract

Stroke is a leading cause of long-term disability worldwide, often resulting in spasticity. Botulinum toxin injections have emerged as a cornerstone in the management of post-stroke spasticity. However, despite their clinical efficacy, maintaining long-term adherence to botulinum toxin therapy remains a significant challenge. This retrospective observational study analyzed 106 patients undergoing botulinum toxin therapy for post-stroke spasticity to identify the key factors influencing treatment continuation. The mean age of the cohort at the time of stroke was 57.7 years, with ischemic strokes accounting for 61.3% of cases and hemorrhagic strokes for 38.7%. A total of 61.3% of patients continued therapy, while 38.7% discontinued therapy due to a variety of reasons. The most common reasons included logistical barriers (43.9%) and comorbidities (36.6%), followed by perceived lack of benefit (24.4%) and clinical resolution (12.2%). Among those citing a lack of benefit, muscular fibrosis was a notable contributor. In the multivariable Cox regression analysis, logistical challenges, such as access to healthcare facilities and administrative difficulties, were associated with discontinuation (HR = 13.95, 95% CI: 5.57–34.94, *p* < 0.001). Comorbidities also significantly increased the likelihood of discontinuation (HR = 3.51, 95% CI: 1.56–7.87, *p* = 0.002), as did the lack of benefit (HR = 14.34, 95% CI: 5.65–36.38, *p* < 0.001) and condition resolution (HR = 19.20, 95% CI: 5.58–66.02, *p* < 0.001). In contrast, demographic and clinical factors, including age at the time of stroke, gender, stroke type, affected side, and baseline spasticity severity, did not significantly influence treatment continuation. These findings underscore the importance of addressing logistical barriers and mitigating the burden of comorbidities to enhance treatment adherence. A shift toward patient-centered approaches that integrate robust rehabilitation services and streamline healthcare accessibility is critical for optimizing outcomes.

## 1. Introduction

Stroke is a major global health challenge, with an estimated 93.8 million people affected worldwide in 2021, including 11.9 million new cases. It ranks as the third leading cause of death, accounting for 7.3 million fatalities, 10.7% of all deaths globally. Despite considerable advancements in medical science, stroke continues to be a leading cause of long-term disability, severely impacting individuals’ quality of life and functional independence, contributing 160.5 million disability-adjusted life-years (DALYs), which represent 5.6% of all DALYs globally [1].

Spasticity has traditionally been defined as a motor disorder marked by a velocity-dependent increase in tonic stretch reflexes (commonly referred to as muscle tone), often accompanied by exaggerated tendon reflexes. This condition arises from the hyperexcitability of the stretch reflex mechanism [2,3]. Years later, Pandyan et al. [4] expanded this understanding by introducing the concept of spasticity as a sensory–motor disorder, highlighting the interplay between sensory and motor components in its pathophysiology [5].

Spasticity is often accompanied by an inability to relax muscles (spastic dystonia) and other positive signs associated with the upper motor neuron syndrome [5]. Post-stroke spasticity is a common complication, with prevalence rates ranging from 4% to 27% within the first six weeks following a stroke, 19% at three months, 21.7% to 42.6% at four to six months, and 17% to 38% at one year [6,7,8], significantly hindering stroke survivors’ daily activities and posing challenges to their recovery and independence [6,7]. While the prevalence estimates of post-stroke spasticity range between 4% and 42.6%, the prevalence of disabling spasticity is reported to vary from 2% to 13% [8].

These figures underscore the critical need for effective interventions to mitigate the impact of spasticity on stroke survivors and enhance their overall quality of life [9]. Among the therapeutic interventions available, botulinum toxin injections have become a cornerstone in the management of post-stroke spasticity [10,11,12]. Botulinum toxin, derived from *Clostridium botulinum*, is a neurotoxin that temporarily inhibits the release of acetylcholine at the neuromuscular junction, leading to localized muscle relaxation [13]. This mechanism of action makes it particularly effective in reducing spasticity [14]. Despite these advantages, the long-term success of botulinum toxin therapy is often hindered by challenges in maintaining continued treatment [9].

Discontinuation of botulinum toxin therapy is a multifaceted issue influenced by a range of factors, including patient-related variables and perceived lack of benefit, with organizational challenges and logistical barriers further contributing to treatment discontinuation [15]. Understanding these determinants is critical for optimizing clinical outcomes, improving resource allocation, and ensuring sustained patient engagement in therapy.

However, despite the importance of the discontinuation of botulinum toxin treatment, this topic remains underexplored in the existing scholarly literature. Only a relatively limited body of research has, indeed, focused explicitly on the treatment of post-stroke spasticity using botulinum toxin, particularly in terms of evaluating its long-term efficacy [15,16,17,18,19]. Most existing studies tend to concentrate on shorter-term outcomes, leaving a significant gap in understanding the sustained benefits and potential drawbacks of botulinum toxin over extended periods. This gap persists whether the treatment is administered as a standalone intervention or in conjunction with physical therapy modalities aimed at enhancing motor function and reducing muscle tone abnormalities [20]. While some studies [15,16,17,18,19] provide some insight into this area, the overall evidence base remains sparse. The majority of studies that explore the long-term management of spasticity tend to focus on broader, mixed etiologies rather than isolating post-stroke spasticity as a distinct condition [21,22,23,24]. Such studies often include participants with spasticity resulting from various neurological disorders, limiting the applicability of their findings to individuals recovering from stroke. This lack of specificity underscores the need for more targeted research to evaluate the long-term outcomes of botulinum toxin therapy specifically for post-stroke spasticity.

This study seeks to fill in this knowledge gap by examining the factors associated with the continued receipt of botulinum toxin injections among stroke patients. Through the use of descriptive statistics, Kaplan–Meier survival analysis, and Cox regressions, we aim to identify the demographic, clinical, and logistical factors that influence treatment retention. The findings are anticipated to provide valuable insights into the reasons underlying treatment discontinuation and offer guidance for developing patient-centered management strategies to maximize the benefits of botulinum toxin therapy. These insights could ultimately help refine clinical protocols and enhance the overall quality of care for individuals living with post-stroke spasticity.

## 2. Results

After excluding 21 patients who had died, a total of 106 patients were included in the analysis (Table 1). The mean age at the time of stroke was 57.7 ± 11.5 years (median: 58.1 years, interquartile range [IQR]: 17.0 years). The cohort consisted of 39 females (36.8%) and 67 males (63.2%). Stroke lesion types were categorized as hemorrhagic (*n* = 41, 38.7%) and ischemic (*n* = 65, 61.3%). In terms of the affected side, 49 patients (46.2%) had left-side involvement while 57 patients (53.8%) had right-side involvement, including one case of bilateral involvement. The median number of toxin injections was eight (IQR: 9.8). The distribution of patients according to the number of toxin injections administered is shown in Table 2, revealing a notable variability and indicating that the highest frequency was observed at three injections, involving 11 patients (10.4% of the cohort). Similarly, five and seven injections were relatively common, reported in nine (8.5%) and eight patients (7.5%), followed by one, six, twelve, and thirteen injections, each recorded in six patients (5.7%). Lower frequencies were observed as the number of injections increased, with certain counts, such as 18, 22, 25, and 27 injections, documented in only one patient each (0.9%). Other intermediate injection frequencies, such as four, eight, and ten, were reported in five patients each (4.7%). Similarly, 19 injections were documented in 5 patients (4.7%). Two and 20 injections were recorded in four patients (3.8%). Three patients (2.8%) were reported in the groups receiving 11, 14, 17, and 26 injections. Finally, nine, 15, 16, 21, and 24 injections were documented in two patients (1.9%). The mean time from stroke onset to injection was 1659 ± 2131 days (median: 656 days, IQR: 2023 days). The treatment duration was, on average, 1368 ± 1055 days (median: 1139 days, IQR: 1691 days).

Of the 106 patients included in this study, 65 (61.3%) were still undergoing treatment at the time of analysis, while 41 (38.7%) had discontinued for various reasons. The most common reason for discontinuation was organizational or logistical challenges related to patient transportation (*n* = 18, 43.9%), followed by comorbidities (*n* = 15, 36.6%; four cases of severe cognitive decline, three instances of malignancies, two cases of lung disease, two cases of COVID-19, one case of cardiovascular disease, one case of vascular parkinsonism, one case of post-traumatic encephalopathy, and one case of depression). Lack of benefit was reported by 24.4% of those who discontinued (*n* = 10), with muscle contracture (muscle shortening detected as a passive range of motion reduction) being the primary issue in seven cases.

Additionally, condition resolution accounted for 12.2% (*n* = 5) of discontinuations. Among the discontinuation cases, 78.0% (*n* = 32) had a single reason for stopping treatment, while 22.0% (*n* = 9) cited multiple reasons. Notably, cases involving multiple reasons frequently combined logistical difficulties with comorbidities, highlighting the complex interplay of factors that impact treatment adherence.

**Figure 1:**
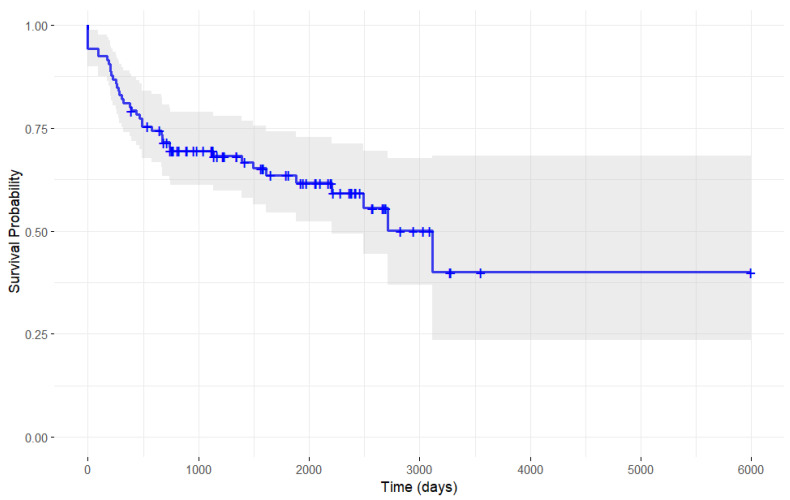
Single-arm Kaplan–Meier analysis of botulinum toxin discontinuation probability in stroke patients.

The Kaplan–Meier analysis (Figure 1) shows that the largest declines in continuation probability occurred during the early phases of treatment, as evidenced by the steep initial drops in the curve, reflecting a higher rate of post-stroke spasticity patients discontinuing botulinum toxin therapy. In contrast, the rate of discontinuation diminished over time, with the continuation curve flattening toward the later stages of treatment, indicating that fewer patients discontinued therapy during this period.

**Figure 2:**
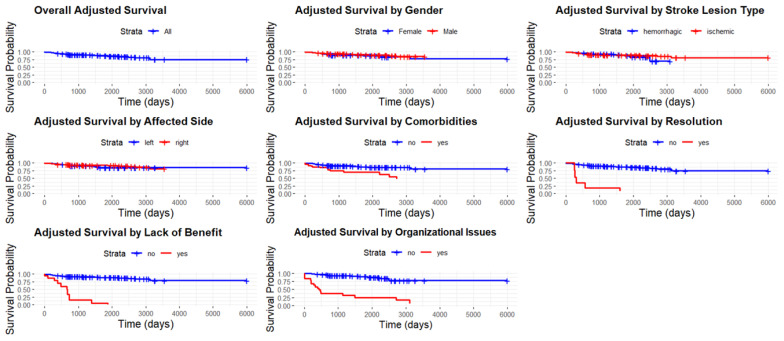
Adjusted survival curve, overall and stratified by gender, for stroke lesion type, affected side, comorbidities, clinical resolution, lack of benefit, and comorbidities.

The Cox regression model demonstrated a satisfactory fit, with a concordance index of 0.879 (standard error: 0.025) and an R-squared value of 0.548. As presented in Table 3 and Figure 2, age at the time of stroke did not significantly influence the likelihood of continuing botulinum toxin injections, as evidenced by both the univariable model (hazard ratio, HR = 1.01, 95% confidence intervals, CI: 0.98–1.04, *p* = 0.499) and the multivariable model (HR = 1.01, 95% CI: 0.98–1.04, *p* = 0.393). Similarly, gender showed no significant effect. Males had an HR of 0.99 (95% CI: 0.52–1.88, *p* = 0.977) in the univariable analysis and an HR of 0.76 (95% CI: 0.33–1.77, *p* = 0.524) in the multivariable model, both non-significant. The type of stroke lesion (ischemic versus hemorrhagic) was also not significantly associated with continued injections. The univariable analysis reported an HR of 1.09 (95% CI: 0.57–2.08, *p* = 0.791), while the multivariable model showed an HR of 0.92 (95% CI: 0.40–2.11, *p* = 0.843). Similarly, the affected side of the stroke (right/bilateral versus left) did not influence the likelihood of continuing injections. The univariable analysis yielded an HR of 1.36 (95% CI: 0.73–2.53, *p* = 0.336), while the multivariable model produced an HR of 0.84 (95% CI: 0.37–1.92, *p* = 0.685). The time from stroke onset to the initiation of botulinum toxin injections was not significantly associated with the likelihood of continuing injections. Both the univariable HR (1.00, 95% CI: 1.00–1.00, *p* = 0.490) and the multivariable HR (1.00, 95% CI: 1.00–1.00, *p* = 0.275) indicated no effect. Similarly, baseline Modified Ashworth Scale (MAS) scores did not significantly influence the outcome, with the univariable analysis reporting an HR of 0.97 (95% CI: 0.91–1.04, *p* = 0.430) and the multivariable analysis showing an HR of 0.93 (95% CI: 0.85–1.02, *p* = 0.118). In contrast, comorbidities, organizational–logistic issues, the absence of benefits, and clinical resolution were significantly associated with discontinued botulinum toxin injections. Comorbidities had an HR of 5.69 (95% CI: 2.99–10.83, *p* < 0.001) in the univariable analysis and 3.51 (95% CI: 1.56–7.87, *p* = 0.002) in the multivariable model. Organizational–logistic issues were associated with an HR of 7.21 (95% CI: 3.84–13.56, *p* < 0.001) in the univariable analysis and an HR of 13.95 (95% CI: 5.57–34.94, *p* < 0.001) in the multivariable model. The absence of benefit showed an HR of 4.93 (95% CI: 2.36–10.27, *p* < 0.001) in the univariable analysis and 14.34 (95% CI: 5.65–36.38, *p* < 0.001) in the multivariable model. Finally, the clinical resolution had an HR of 4.56 (95% CI: 1.75–11.86, *p* = 0.002) in the univariable analysis and 19.20 (95% CI: 5.58–66.02, *p* < 0.001) in the multivariable model.

## 3. Discussion

This present study provides valuable insights into the factors influencing botulinum toxin treatment discontinuation in post-stroke spasticity. Despite the well-documented efficacy of this treatment in managing spasticity [25,26,27], a few studies have specifically explored the determinants of its discontinuation among stroke patients. By contextualizing our results within this limited body of research, we aim to contribute to a deeper understanding of this clinically important issue.

Our study highlights high dropout rates, particularly during the early years of treatment. This finding is consistent with the existing scholarly literature. For instance, the study by Falcone et al. [16] reported that nearly 50% of dropouts occurred within the first four years, often due to unmet patient expectations. Similarly, we observed that treatment adherence declined most sharply in the initial phase, primarily due to organizational challenges.

Moreover, demographic and clinical factors—including age, gender, type of stroke lesion, side of involvement, or time from stroke onset to the initiation of injections—were not significantly associated with treatment continuation. This aligns with the findings of Cinone et al. [15], who also found that these factors played a limited role in predicting adherence to botulinum toxin therapy, suggesting that, instead, logistical barriers—such as access to healthcare facilities, availability of caregiver support, and administrative challenges—are more influential in determining treatment adherence. More in detail, Cinone et al. [15] reported that logistical issues accounted for 37.5% of treatment discontinuations, a trend that our study also confirmed.

However, the same study [15] found that severe spasticity might predict earlier discontinuation due to the need for alternative interventions, while we were not able to find any significant correlation between MAS scores and treatment adherence. This discrepancy underscores the multifactorial nature of treatment continuation and suggests that the relationship between spasticity severity and adherence may be complex and context-dependent. Such a relationship could be mediated or compounded by non-clinical variables, which, though often overlooked, can have a greater impact on treatment adherence than clinical factors. For example, Santamato et al. [22] examined the effects of the COVID-19 pandemic on botulinum toxin therapy and highlighted the negative consequences of treatment discontinuation on patient independence, quality of life, and perceived spasticity. Our study similarly found that logistical barriers, exacerbated by systemic inefficiencies, play a decisive role in treatment adherence, often outweighing clinical considerations. Additionally, both our findings and those of Santamato et al. [22] underscore the critical role of rehabilitation services. While Santamato et al. [22] demonstrated that a lack of rehabilitation services was significantly associated with worsening independence, our study points to the broader need for systemic improvements to enhance accessibility and patient retention.

Beyond logistical barriers, comorbidities emerged as the second most significant factor contributing to treatment discontinuation. However, none of the 15 patients who discontinued treatment due to comorbidities had conditions that, in themselves, contraindicated therapy. Specifically, no patient presented with myasthenia gravis, infections, or inflammatory conditions at the injection site—conditions that, to the best of our knowledge, are the only absolute contraindications to botulinum toxin treatment. Instead, the observed comorbidities included serious illnesses such as cancer, chronic conditions, cardiac diseases, and cerebrovascular disorders, which led to a general decline in health and a reduced prioritization of spasticity treatment. Additionally, these health issues introduced logistical complexities, particularly in arranging patient transportation. Many of these patients were eventually transferred to healthcare facilities, highlighting the interplay between comorbidities and logistical challenges. In other words, comorbidities not only influenced treatment discontinuation but also compounded the logistical difficulties of patient management, reinforcing the pivotal role of healthcare accessibility in long-term treatment adherence.

Taken together, these findings advocate for a patient-centered approach to spasticity management, integrating robust rehabilitation services with efforts to mitigate logistical and administrative barriers. The issue of logistical barriers, mainly caused by patient transportation, can potentially be resolved through the administration of botulinum toxin therapy at home, with the assistance of portable ultrasound devices. This approach would not only enhance accessibility for patients with mobility challenges but also improve adherence to treatment regimens and overall therapeutic outcomes. Moreover, leveraging telemedicine for follow-up assessments and interdisciplinary consultations can further streamline care delivery, reducing the burden on both patients and healthcare providers [28,29].

## 4. Strengths and Limitations

This study offers several notable strengths. First, it leverages a robust methodological approach, combining descriptive statistics, Kaplan–Meier analysis, and Cox regressions, to identify factors influencing the continuation of botulinum toxin therapy in stroke patients. The comprehensive inclusion of both univariable and multivariable models ensures a thorough evaluation of potential predictors, providing a nuanced understanding of the interplay between demographic, clinical, and systemic factors. Additionally, the dataset’s focus on post-stroke patients—a population at high risk of spasticity—enhances the clinical relevance of the findings. This study’s emphasis on organizational and logistic barriers, often overlooked in similar research, represents an important contribution, shedding light on the non-clinical determinants of treatment adherence. Finally, the integration of actionable insights into healthcare delivery challenges offers practical value for clinicians and policymakers aiming to improve patient outcomes.

However, this study is not without limitations. The single-center design and the sample size, although adequate for initial exploration, may limit the generalizability of the findings, especially across different healthcare settings or populations. Furthermore, only *Incobotulinum* toxin A was assessed, which may not capture potential differences in outcomes associated with other botulinum toxin formulations. Moreover, the observational nature of this study precludes causal inferences and unmeasured confounders, such as patient attitudes toward treatment, caregiver support, and socioeconomic factors, which may influence the results. Additionally, the reliance on retrospective data introduces potential biases, including recall and selection bias, which could affect the accuracy of reported reasons for treatment discontinuation. Further, this study does not explore the impact of specific rehabilitation programs or variations in healthcare delivery systems, which could provide deeper insights into systemic influences on treatment adherence, for which multi-center, comparative studies would be needed. Finally, although the MAS is widely used, it is a single measure that may not fully capture the complexity of spasticity severity, potentially underestimating its impact on treatment discontinuation.

## 5. Conclusions

This study provides valuable insights into the factors influencing the continuation of botulinum toxin therapy in post-stroke patients with spasticity. While demographic and clinical variables, such as age, gender, stroke lesion type, affected side, time from stroke onset to the initiation of injections, and spasticity severity, were not significantly associated with treatment adherence, logistical and organizational challenges as well as comorbidities emerged as pivotal contributors to treatment discontinuation. These findings highlight the need to shift focus from purely clinical predictors to addressing broader healthcare delivery challenges to enhance patient retention and optimize outcomes. The results underscore the importance of a patient-centered, holistic approach to managing post-stroke spasticity, which considers not only clinical needs but also social, logistical, and organizational barriers. Future research should delve deeper into unmeasured factors, such as patient and caregiver perceptions, socioeconomic influences, and healthcare accessibility, to develop targeted strategies that mitigate barriers to long-term treatment adherence. By addressing these issues, healthcare systems can better support patients in maintaining treatment continuity and achieving improved functional outcomes [30].

## 6. Material and Methods

### 6.1. Ethical Clearance

The study protocol TOXIN_UMNS received ethical clearance from the Liguria Regional Ethics Committee during its session held on September 9, 2024. Authorization was granted under Deliberation No. 1924, dated December 12, 2024, by the IRCCS Ospedale Policlinico San Martino, Genoa, Italy.

This retrospective observational study is part of a series investigating patients with post-stroke spasticity undergoing botulinum toxin treatment. While this study specifically examines the determinants of treatment discontinuation, the other studies in the series focus on the long-term effectiveness of the therapy as well as temporal trends and shifts in the administration of botulinum toxin.

### 6.2. Patient Selection and Recruitment

Patients were recruited from a single-center outpatient spasticity clinic in northern Italy (IRCCS Ospedale Policlinico San Martino, Genoa, Italy), one of the largest centers in Italy administering the botulinum toxin. This center manages approximately 140 patients in subacute care and 10 patients referred from the surrounding territory, 20% of whom underwent botulinum toxin injections. Additionally, the clinic follows a cohort of chronically managed patients who receive ongoing care and treatment. 

Eligibility required a confirmed clinical diagnosis of post-stroke spasticity, an age of 18 years or older at the time of recruitment, and a history of at least one botulinum toxin injection for spasticity following the stroke incident. The botulinum toxin administered was *Incobotulinum* toxin A.

Written informed consent was obtained from all participants before inclusion in this study. Patients with incomplete medical records and those with other neurological conditions unrelated to stroke were excluded. Recruitment was conducted during routine outpatient visits from January 2013 to December 2023, and baseline demographic, clinical, and treatment-related data were collected to ensure a diverse and representative cohort. The study population was further stratified by variables such as stroke type, affected side, and time from stroke onset to injection to facilitate detailed subgroup analyses.

### 6.3. Statistical Analysis

Descriptive statistics were performed, including the calculation of means and standard deviations for continuous variables and percentages for categorical variables. Visualization of the data was also conducted to aid in understanding the distribution and relationships within the dataset. A Kaplan–Meier analysis was performed to estimate survival functions and visualize the probability of continued botulinum toxin injections over time. A Cox analysis was employed to evaluate factors associated with the continued receipt of botulinum toxin injections in stroke patients, with both univariable and multivariable models. Variables under study included age at the time of stroke, gender, stroke lesion type (ischemic versus hemorrhagic), affected side of the stroke (right/bilateral versus left), time from stroke onset to the initiation of injections, the severity of spasticity as assessed by the MAS, and the reason for discontinuation (comorbidities, logistical issues, lack of benefit, and clinical resolution). The reasons for discontinuation were ascertained through a combination of methods, including a thorough review of medical records and conducting patient interviews. The fit of the Cox regression model was assessed using the concordance index, which quantifies the predictive accuracy of the model. A higher concordance index indicates a better fit. Additionally, the model’s explanatory power was evaluated using the R-squared value. The proportional hazards assumption was tested using Schoenfeld residuals to ensure the validity of the model assumptions.

All statistical analyses and data visualizations were performed using the R programming environment (R 4.2.3 Core Team, Vienna, Austria), with a significance level set at *p* < 0.05. The results were reported with HRs and 95% CIs to quantify associations and assess the robustness of findings. 

## Figures and Tables

**Table 1:** Demographics of the study cohort. Abbreviations: IQR (interquartile range); SD (standard deviation).

Variable	Value (n = 106)
Mean Age (years)	Mean ± SD: 57.7 ± 11.5 (Median: 58.1, IQR: 17.0)
Gender	Female: 39 (36.8%) Male: 67 (63.2%)
Stroke Type	Ischemic: 65 (61.3%) Hemorrhagic: 41 (38.7%)
Affected Side	Left: 49 (46.2%) Right: 57 (53.8%)
Number of Toxin Injections	Median: 8, IQR: 9.8
Time from Stroke to Injection (days)	Mean ± SD: 1658.9 ± 2130.8 (Median: 656, IQR: 2023)
Patients Continuing Treatment	65 (61.3%)
Patients Discontinuing Treatment	41 (38.7%)
Reasons for Discontinuation	
Logistical Barriers	18 (43.9%)
Comorbidities	15 (36.6%)
Perceived Lack of Benefit	10 (24.4%)
Clinical Resolution	5 (12.2%)

**Table 2:** Number of toxin injections completed by each patient.

Number of Toxin Injections	Number (Percentage) of Patients
1	6 (5.7%)
2	4 (3.8%)
3	11 (10.4%)
4	5 (4.7%)
5	9 (8.5%)
6	6 (5.7%)
7	8 (7.5%)
8	5 (4.7%)
9	2 (1.9%)
10	5 (4.7%)
11	3 (2.8%)
12	6 (5.7%)
13	6 (5.7%)
14	3 (2.8%)
15	2 (1.9%)
16	2 (1.9%)
17	3 (2.8%)
18	1 (0.9%)
19	5 (4.7%)
20	4 (3.8%)
21	2 (1.9%)
22	1 (0.9%)
24	2 (1.9%)
25	1 (0.9%)
26	3 (2.8%)
27	1 (0.9%)

**Table 3:** Findings from the univariable and multivariable Cox regression analyses.

Variable	HR (Univariable)	HR (Multivariable)
Age at the time of stroke	1.01 (0.98–1.04, *p* = 0.499)	1.01 (0.98–1.04, *p* = 0.393)
Gender (male vs. female)	0.99 (0.52–1.88, *p* = 0.977)	0.76 (0.33–1.77, *p* = 0.524)
Stroke lesion type (ischemic vs. hemorrhagic)	1.09 (0.57–2.08, *p* = 0.791)	0.92 (0.40–2.11, *p* = 0.843)
Affected side (right vs. left)	1.36 (0.73–2.53, *p* = 0.336)	0.84 (0.37–1.92, *p* = 0.685)
Time from stroke to injection	1.00 (1.00–1.00, *p* = 0.490)	1.00 (1.00–1.00, *p* = 0.275)
Baseline MAS	0.97 (0.91–1.04, *p* = 0.430)	0.93 (0.85–1.02, *p* = 0.118)
Comorbidities	5.69 (2.99–10.83, *p* < 0.001)	3.51 (1.56–7.87, *p* = 0.002)
Organizational-logistic issues	7.21 (3.84–13.56, *p* < 0.001)	13.95 (5.57–34.94, *p* < 0.001)
Lack of clinical benefit	4.93 (2.36–10.27, *p* < 0.001)	14.34 (5.65–36.38, *p* < 0.001)
Clinical resolution	4.56 (1.75–11.86, *p* = 0.002)	19.20 (5.58–66.02, *p* < 0.001)

## Data Availability

The original contributions presented in this study are included in the article. Further inquiries can be directed to the corresponding authors.
